# A Giant Magneto-Strictive Material-Based Fabry–Perot Interferometer-Type 3D Vector Magnetic Field Sensor

**DOI:** 10.3390/nano16050323

**Published:** 2026-03-04

**Authors:** Ze Yu, Dongran Liu, Chunbo Su, Yingjie Qiao, Xiaodong Wang, Tao Geng

**Affiliations:** 1 Key Laboratory of Superlight Materials and Surface Technology, Ministry of Education, College of Materials Science and Chemical Engineering, Harbin Engineering University, Harbin 150001, China; yuze1996@hrbeu.edu.cn (Z.Y.); qiaoyingjie@hrbeu.edu.cn (Y.Q.); 2 Key Lab of In-Fiber Integrated Optics, Ministry Education of China, Harbin Engineering University, Harbin 150001, China; capybaba777@hrbeu.edu.cn (D.L.); cbsu_20@hrbeu.edu.cn (C.S.)

**Keywords:** vector magnetic field sensor, giant magneto-strictive material, Fabry–Perot interferometers

## Abstract

This paper presents the design and experimental validation of a highly sensitive vector magnetic field sensor based on three mutually orthogonal Fabry–Perot interferometers (FPIs). The orthogonally arranged FPIs are bonded to a giant magneto-strictive material (GMM) block. Under an applied magnetic field, the magneto-strictively induced strain in the GMM block is transferred to the FPIs. Meanwhile, the FPIs, composed of single-mode fiber (SMF)–hollow-core fiber (HCF)–SMF, are further modulated by CO_2_ laser, by which the higher sensitivities are obtained. The highest sensitivities of FPIs achieved 245.13, 159.06, and 168.59 pm/mT on the X-Y, X-Z, and Y-Z planes, respectively. By demodulating the distinct wavelength drifts of the three orthogonal FPIs, both the magnitude and direction of the magnetic field can be simultaneously determined.

## 1. Introduction

Magnetic field measurement [1] is a cornerstone of physics and engineering, enabling safety and reliability in both fundamental research and practical applications [2]. While scalar sensors (measuring only field strength) are widely used [3], vector magnetic field measurement, which captures both strength and direction, is more reliable for revealing the full “shape” of magnetic fields. Usually, scalar magnetic field measurement is easier to realize and many a sensor based on electronic [4] or optic [5] principles have been demonstrated. Compared with magnetic field sensors based on electronic devices, fiber-optic sensors [6] have the advantages of high sensitivity, compact volume and anti-electromagnetic interference ability. As a result, fiber-optic magnetic field sensors have been increasingly investigated over the past decade.

Fiber-optic magnetic field sensors, categorized by their working mechanisms, can be divided into two main types: magneto-optic effect-based [7] and magneto-strictive material-based [8]. For the magneto-optic effect-based sensors, the working principle relies on the refractive index change induced by the magnetic fluid [9] or magneto-photonic crystal. Then, the wavelength or intensity of the refractive index-sensitive fiber structure will shift to the magnetic field change. In order to realize vector magnetic field measurement, the fiber structure needs to be designed in a non-circular symmetrical form [10] or rely on special types of fiber [11]. For example, Wang et al. proposed an open-cavity Mach–Zehnder interferometer (MZI) [12], which has the ability to distinguish the direction of the magnetic field by demodulating the different degrees of wavelength shifts and where the highest sensitivity reaches −17.306 nm/mT. Guo et al. designed an MZI [13] based on special fiber splicing. Due to the circular asymmetry of the structure, their sensor also has the ability to distinguish the direction of the magnetic field. In addition, many studies [14,15,16] focus on improving sensitivity by splicing special fibers or tapering the structure and realizing vector magnetic field measurement by breaking the circular symmetry. However, when these sensors operate under real-world magnetic field conditions they still fail to accurately determine the magnetic field direction, merely exhibiting varying sensitivities across different magnetic field orientations. Also, the magnetic fluid has a limited shelf life. As such, their magneto-optic coefficients change over just a few months, which results in significant challenges to sensor recalibration.

In recent years, fiber-optic sensors based on GMMs [17,18] have garnered increasing attention. This growing interest is attributed to their advantages, including long-term stability and rapid response speed. The operating mechanism involves converting the magnetic field into structural stress, which is then transferred to the optical fiber structure bonded onto the material. For instance, Wang et al. realized highly sensitive scalar magnetic field measurement by combining the Vernier effect and a GMM [19]. However, the previously reported studies focus on scalar sensing and the problem of realizing rapid measurement for both magnetic field direction and intensity.

In this paper, a vector magnetic field sensor based on combining a Terfenol-D block (the highest magneto-striction coefficient of GMMs) and three mutually orthogonal FPIs is proposed and experimentally testified. With a fiber cleaving and splicing technique, all-fiber FPIs are prepared by splicing SMFs and HCFs. Meanwhile, with a laser polishing technique, the sensitivity of the FPIs are improved. Due to the magneto-strictive effect, the Terfenol-D generates axial deformation aligned with the magnetic field direction under an applied magnetic field, thereby inducing strain in the optical fiber structure bonded to it. When the sensor operates within an unknown magnetic field, the three mutually orthogonal FPIs exhibit varying degrees of wavelength drift, thereby enabling the simultaneous determination of both the magnetic field direction and intensity. The experimental results present that the sensitivities of the FPIs are obtained as 245.13 pm/mT, 159.06 pm/mT, and 168.59 pm/mT in the X-Y, X-Z, and Y-Z planes in the range of 0–11.2 mT, respectively. Also, the sensor has the ability to distinguish the direction of the magnetic field. In addition, the sensor is temperature insensitive, which brings about another advantage for magnetic field sensing. In actual experiments the sensor possesses considerable stability and repeatability, and the maximum relative errors of magnetic field angle and intensity are obtained as 1.50% and 0.74%, respectively. The sensor’s EMI immunity and compact 3D orthogonal structure enable reliable vector magnetic field sensing in extreme environments where electronic sensors fail. Key applications include high-voltage transformer monitoring, MRI gradient field mapping, and aerospace or underwater navigation in confined spaces. It is believed that the proposed sensor has the potential to be applied in various scenario for vector magnetic field measurement and brings about a fresh thinking to solve such problems.

## 2. Working Principle

**Figure 1:**
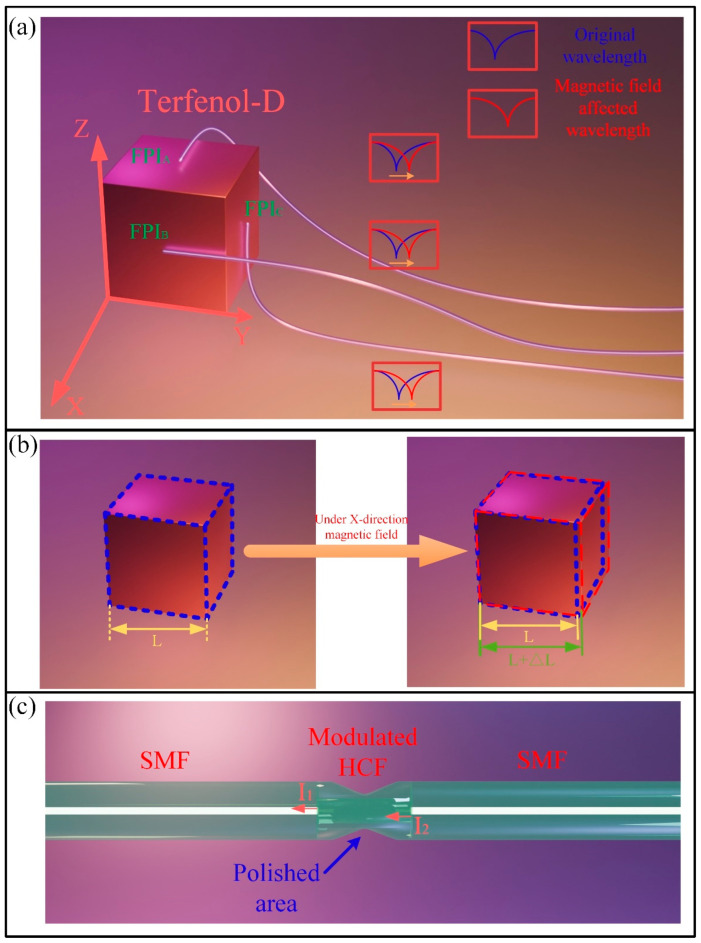
(**a**) Schematic diagram of the proposed sensor. (**b**) Converted strain in transverse directions under external magnetic field. (**c**) Schematic diagram of proposed FPI.

Figure 1a illustrates the overall structure of the proposed sensor. To achieve vector magnetic field measurement, the fiber structure is bonded to the Terfenol-D block in a mutually orthogonal configuration. To describe the sensor more clearly a 3D coordinate system is established, with the three fiber structures remaining parallel to the X-Y, X-Z, and Y-Z planes, respectively. Meanwhile, the fiber structures parallel to the X-Y, X-Z, and Y-Z planes are defined as FPI_A_, FPI_B_, and FPI_C_, respectively. Here, Terfenol-D is a magneto-strictive material with the highest magneto-strictive coefficient of GMMs [19], exhibiting a magneto-strictive coefficient exceeding ~1600 ppm (Tb (0.4), Dy (0.6), Fe (1.90)). The operating principle of Terfenol-D can be considered as converting external magnetic fields into structural stress. Under the magnetic field environment, the structural strain $\varepsilon_{Terferol-D}$ induced by magnetic field can be expressed as follows [17]:

(1) $$\varepsilon_{Terferol-D}=\gamma B,$$

where γ is the magneto-strictive coefficient of Terfenol-D, and B is the external magnetic field. Under an applied magnetic field, the Terfenol-D exhibits maximum strain along the magnetic field direction [20], while the strains in transverse directions can be resolved, which is illustrated in Figure 1b. Here, the FPI_A_ is parallel to the *X*-axis, FPI_B_ is parallel to the *Y*-axis and FPI_C_ is parallel to the *Z*-axis. Based on the theory, the wavelength shift $\Delta \lambda_{i}$ of each FPI is expressed as follows:

(2) $$\Delta \lambda_{i}=S_{i}B_{i},$$

where $S_{i}$ expresses the corresponding sensitivity of the FPI_A_, FPI_B_, and FPI_C_ at the *X*-axis, *Y*-axis, and *Z*-axis, respectively. In spherical coordinates, the azimuth angle ϕ is defined as the angle between the vector and the *x*-axis within the x-y plane, while the polar angle θ is defined as the angle between the vector and the *z*-axis. According to the decomposition formulas converting spherical coordinates to Cartesian coordinates, the orthogonal components (x, y, z) of the magnetic field, $B_{x}$, $B_{y}$ and $B_{z}$, can be expressed as follows:

(3) $$\left|B\right|=\sqrt{{B_{x}}^{2}+{B_{y}}^{2}+{B_{z}}^{2}},$$

(4) $$\left\{\begin{array}{l} B_{x}=B\cdot \sin\theta \cdot \cos\phi \\ B_{y}=B\cdot \sin\theta \cdot \sin\phi \\ B_{z}=B\cdot \cos\theta \end{array}\right..$$

In addition, it is worth mentioning that the magneto-strictive coefficient of the Terfenol-D material varies in different directions. As a result, the relationship between wavelength shifts and the magnetic field is calculated as follows:

(5) $$\left\{\begin{array}{l} \Delta \lambda_{a}=S_{a}\cdot B_{x}=S_{a}\cdot B\cdot \sin\theta \cdot \cos\phi \\ \Delta \lambda_{b}=S_{b}\cdot B_{y}=S_{b}\cdot B\cdot \sin\theta \cdot \sin\phi \\ \Delta \lambda_{c}=S_{c}\cdot B_{z}\cdot \cos\theta \end{array}\right..$$

where $S_{a}$, $S_{b}$ and $S_{c}$ present the sensitivities of the FPIs. In actual testing, it is necessary to first calibrate the magnetic sensitivity in different directions and then calculate according to Equations (3)–(5). Figure 1c presents the schematic diagram of the fiber structure bonded onto the Terfenol-D. Here, the three mutually orthogonal FPIs are prepared by splicing HCFs between the SMFs. Then, the portion of HCFs are further etched by high-frequency CO_2_ laser, by which the stress distribution of the HCF portion is modulated under the laser etching.

**Figure 2:**
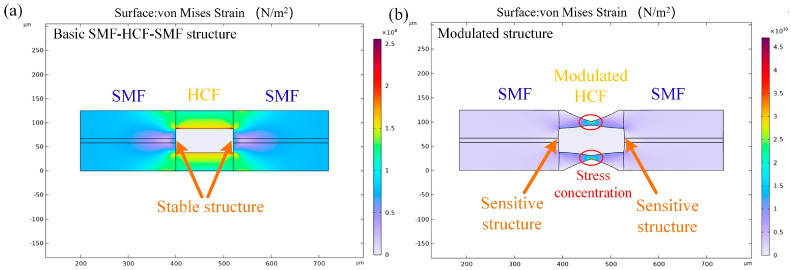
Simulated stress distribution of (**a**) basic structure and (**b**) modulated structure.

For the basic SMF-HCF-SMF structure, the HCF portion is insensitive to strain due to its relatively stable structure. After modulation, the HCF section exhibits enhanced deformability under stress, thereby achieving significantly improved strain sensitivity. Figure 2 illustrates the stress distribution in the unmodified and modulated HCF sections simulated by finite element simulation. Here, the relevant parameters for the simulation are set as: diameter of HCF/SMF: 125 μm (cladding), 10 μm (core, SMF), and 50 μm (air-cavity of HCF); Young’s modulus: 72.5 GPa; material density: 2700 kg/m^3^; and Poisson’s ratio: 0.17. It is proved that under identical stress conditions the modulated structure exhibits significantly greater deformability, which indicates higher sensitivity to strain.

Based on the principle of the fiber-based FPI, the interference within the HCF is simply expressed as follows [21]:

(6) $$I=I_{1}+I_{2}+2\sqrt{I_{1}I_{2}}\cos\phi .$$

(7) $$\phi =\frac{4\pi nL_{FPI}}{\lambda_{FPI}}.$$

where $I_{1}$ and $I_{2}$ presents the intensities of the reflection mirrors of the incident light in HCFs, respectively. n is the refractive index of the air-cavity, $L_{FPI}$ is the length of air-cavity and $\lambda_{FPI}$ is the operating wavelength of the FPI. Meanwhile, the free spectrum range (FSR) of the proposed FPI is determined as follows [22]:

(8) $$FSR_{FPI}=\frac{{\lambda_{FPI}}^{2}}{2nL_{FPI}}.$$

For the proposed structure, when exposed to a magnetic field the Terfenol-D material deforms along the magnetic field direction, causing $L_{FPI}$ to increase. According to Equation (8), a longer $L_{FPI}$ induces a red-shift in $\lambda_{FPI}$. Meanwhile, due to the distinct wavelength drifts of the three mutually orthogonal FPIs, the magnitude and direction of the current magnetic field can be derived using Equation (2).

## 3. Material and Methods

**Figure 3:**
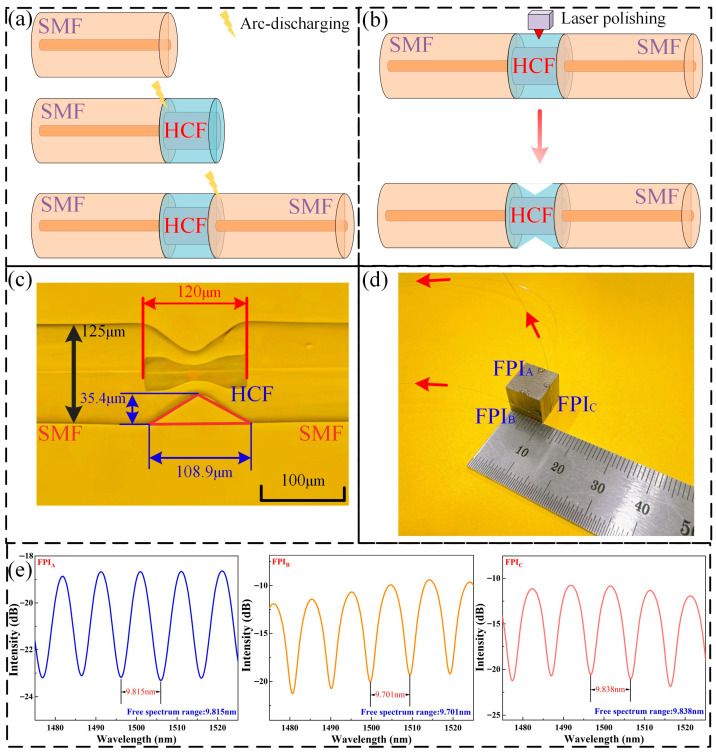
Sensor preparation. (**a**) Preparation of the FPI. (**b**) Process of laser polishing. (**c**) Actual image of the prepared FPIs. (**d**) Actual image of the prepared sensor. (**e**) Reflective spectrum of the three FPIs.

This section introduces the sensor preparation, which is mainly derived into three steps which are presented in Figure 3. Firstly, in order to obtain the proposed FPI, a section of HCFs (air-cavity diameter: 30 μm) is introduced between the SMFs (cladding/core diameter: 125/10 μm), which is presented as Figure 3a. Here, the selection of HCFs with a smaller diameter air-cavity is attributed to the fact that the modulated section within the smaller air-cavity structure is deeper, which enhances the sensitivity to strain. Meanwhile, according to Equation (8), the FSR presents an inverse relationship with $L_{FPI}$. According to the theory, the strain sensitivity is inversely proportional to the length of the cavity for a single cavity structure [23]. As a result, the FPI structure is optimized towards shorter and deeper modulating regions.

Secondly, the prepared FPI is further modulated by CO_2_ laser [24], and this process is presented as Figure 3b. Here, the focused spot diameter of the laser is 100 μm in order to avoid damaging the structure, and the shortest length of HCF is controlled to be 120 μm. Figure 3c presents the actual image of the prepared structure, where the modulated depth and width of the V-shaped groove are measured as 35.4/108.9 μm. It is worth mentioned that the fabricating process for every FPI is controlled in the same way, by which the sensing performance of each FPI can be effectively controlled as almost constant.

Thirdly, the prepared FPIs are bonded to the Terfenol-D block (size: 10 × 10 × 10 mm; magneto-strictive coefficient: ~1600 ppm (X-Y plane). Figure 3d displays the prepared sensor, in which the three FPIs are arranged in an orthogonal configuration across three distinct planes of the Terfenol-D block, with both ends fixed using UV-curable adhesive to ensure they are centered within each plane. Here, each FPI bonded to the Terfenol-D surface is subjected to a specific pre-stress during bonding, thereby preventing its loosening. Figure 3e displays the reflective spectrum of the three FPIs. It is deduced that the FSR and contrast radio of these three FPIs remain almost constant, which facilitates vector magnetic field sensing.

## 4. Experimental Results and Discussion

**Figure 4:**
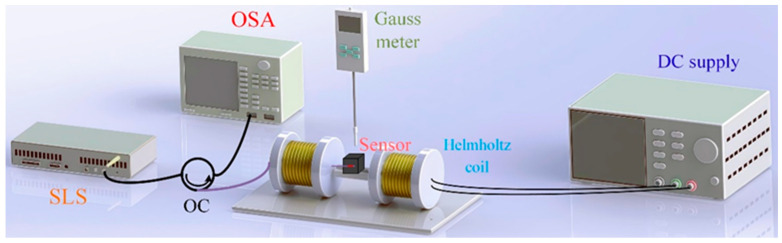
Experimental system for magnetic field characteristics test.

To investigate the magnetic field sensing characteristics of the sensor, the experimental system for magnetic field characteristics shown in Figure 4 is constructed. The system comprises a pair of Helmholtz coils to generate a constant scalar magnetic field, a super-continuum light source (SLS, YSL, SC-5), a circulator, and an optical spectrum analyzer (OSA, YOGAWAGA, AQ6370D) for detecting real-time optical signal variations, and a 3D-printed fixture to support the sensor. Here, the intensity of the scalar magnetic field induced by the Helmholtz coils is adjusted by changing the output current intensity. Before testing the magnetic field characteristic of the sensor, the scalar magnetic field generated by the coil was measured using a Gauss meter, with the magnetic field strength at the center of the coil increasing by 1.12 mT for every 1 A increase in current. The maximum range of the current is 10A, corresponding the maximum magnetic field range of 11.2 mT.

**Figure 5:**
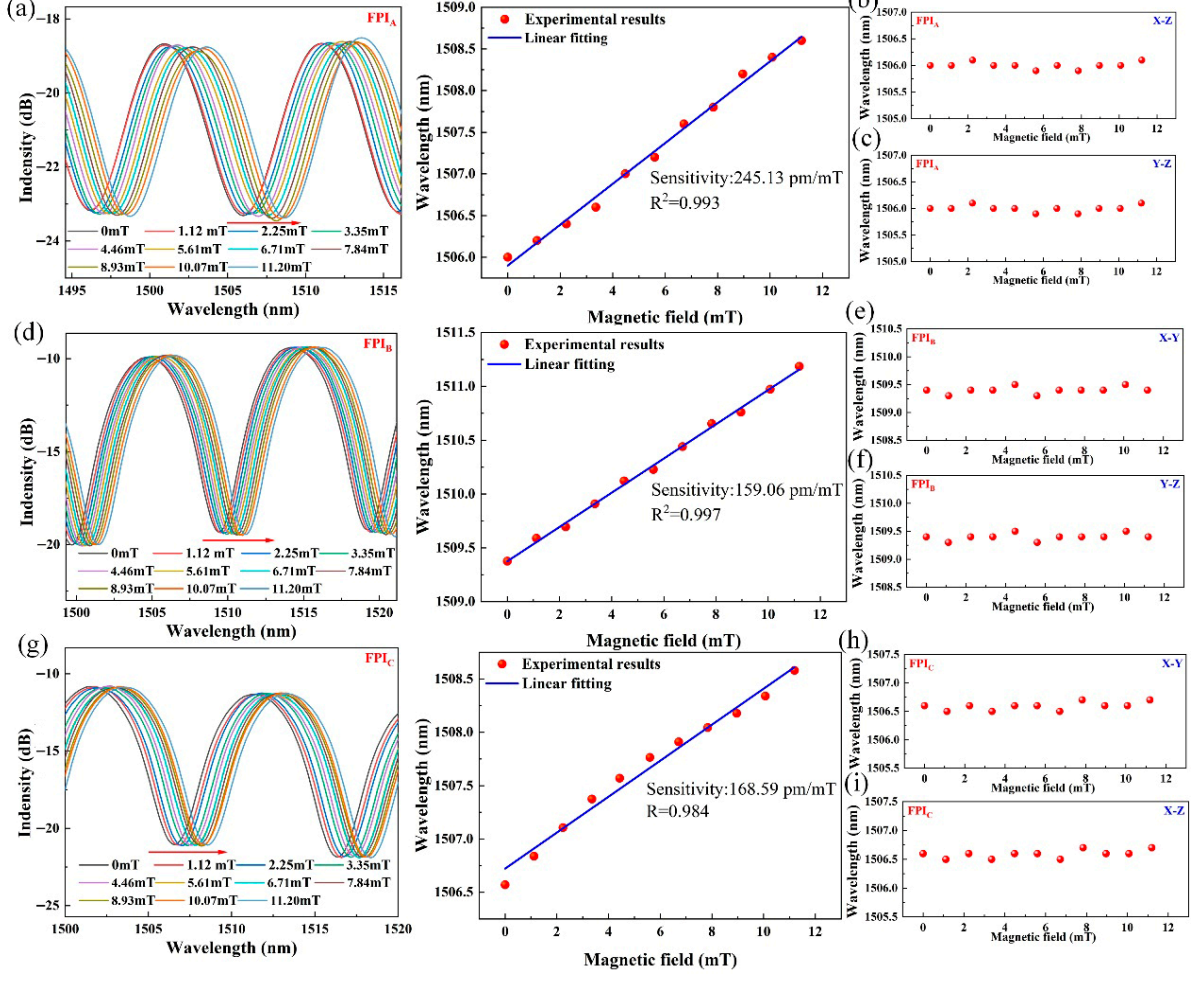
Magnetic field characteristic for three FPIs. Magnetic response of FPIA at (**a**) X-Y plane, (**b**) X-Z plane and (**c**) Y-Z plane. Magnetic response of FPIB at (**d**) X-Z plane, (**e**) X-Y plane and (**f**) Y-Z plane. Magnetic response of FPIC at (**g**) Y-Z plane, (**h**) X-Y plane and (**i**) X-Z plane.

**Figure 6:**
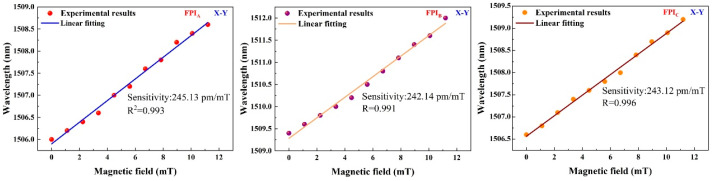
Magnetic field sensitivities of FPIA, FPIB and FPIC at X-Y plane.

**Figure 7:**
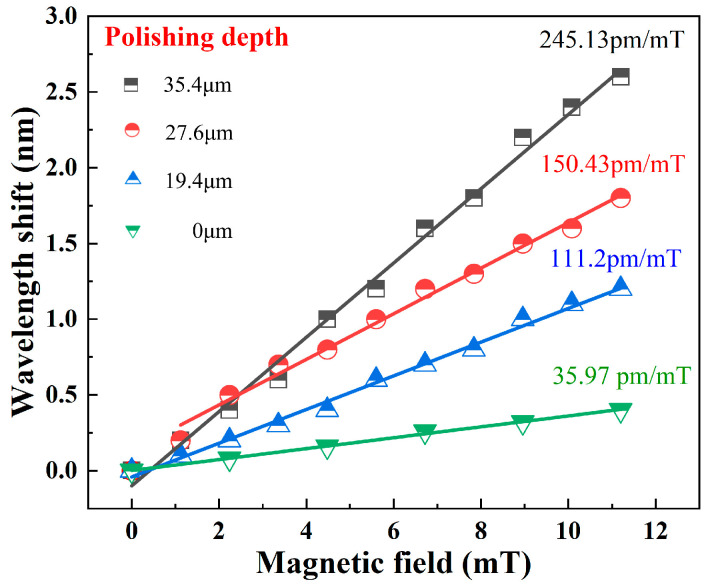
Comparison of sensitivities of FPIs with different polishing depths.

Here, the scalar magnetic field characteristics of three FPIs are first investigated. During the experiment, the magnetic field increases from 0 to 11.2 mT with a step of 1.12 mT. Based on Figure 1b, a 3D coordinate system is defined. When one of the FPIs is aligned with the magnetic field direction, the three FPIs remain parallel to the X-Y, X-Z, and Y-Z planes. Figure 5 presents the magnetic field responses of the three FPIs when the sensor is parallel to the X-Y, X-Z, and Y-Z planes. Figure 5a–c, Figure 5d–f and Figure 5g–i present the magnetic field responses of the FPIA, FPIB, and FPIC when the sensor is placed parallel to the X-Y, X-Z, and Y-Z planes, respectively. It is found that the magnetic field sensitivities of FPIA are obtained as 245.13 pm/mT at the X-Y plane, 0 pm/mT at X-Z plane and 0 pm/mT at Y-Z plane. The sensitivities of FPIB are obtained as 159.06 pm/mT at the X-Z plane, 0 pm/mT at X-Y plane and 0 pm/mT at Y-Z plane. The sensitivities of FPIC are obtained as 168.59 pm/mT at the Y-Z plane, 0 pm/mT at X-Y plane and 0 pm/mT at X-Z plane. Here, the variations in magnetic field sensitivity among the three FPIs primarily stem from the formation of a most sensitive direction in the magneto-strictive material during its fabrication process, where the magneto-strictive effect is more pronounced than in other directions. In addition, since the three FPIs are fabricated using identical procedures and methods, we validated the magnetic field sensitivity of the FPIs on the same surface of Terfenol-D material. This is achieved by fixing the three FPIs on the X-Y plane of the Terfenol-D material and conducting magnetic field sensitivity measurements, with the results presented in Figure 6. The experimental results show that the sensitivities of the FPIA, FPIB and FPIC at the X-Y plane are obtained as 245.13 pm/mT, 242.14 pm/mT and 243.12 pm/mT, respectively. It demonstrates that magneto-strictive materials exhibit anisotropy, where the magneto-strictive coefficient in non-sensitive directions is smaller than that in sensitive directions, which is consistent with the theoretical analysis. Figure 7 presents the magnetic field sensing characteristics of sensing FPIs with different polishing depths of 0 µm, 19.4 µm, 27.6 µm and 35.4 µm. Within the measuring range of 0–11.2 mT, the sensitivity of the sensor with a polishing depth of 35.4 µm achieves 245.13 pm/mT, which is 6.81 times stronger than the above-mentioned single FPI.

**Figure 8:**
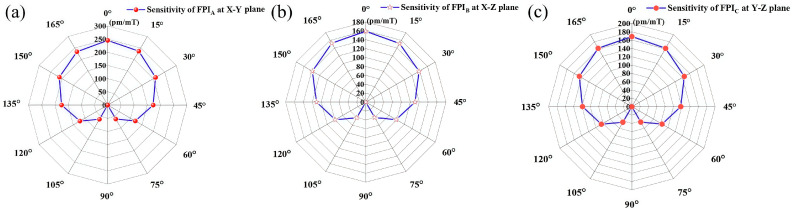
Magnetic field response of (**a**) FPIA at the X-Y plane, (**b**) FPIB at the X-Z plane, and (**c**) FPIC at the Y-Z plane.

Next, in order to realize vector magnetic field measurement, the magnetic field responses of three FPIs at different angles in the X-Y, X-Z, and Y-Z planes are tested, and the results are presented in Figure 7. Here, the sensor is firstly fixed at the X-Y plane, and the wavelength response to the magnetic field of FPIA is measured each 15° in the range of 0–180° by adjusting the Terfenol-D material. Here, the 0 degree direction on each surface is parallel to the positive directions of the X, Y, and Z axes. Figure 8a presents the sensitivities of FPIA at different angles in the X-Y plane, Figure 8b presents the sensitivities of FPIB at different angles in the X-Z plane, and Figure 8c presents the sensitivities of FPIC at different angles in the Y-Z plane. From the experimental results, it can be inferred that each FPI in the sensor is capable of resolving the magnetic field angle within its host plane. When the sensor is exposed to an unknown magnetic field, the three FPIs produce wavelength drifts with distinct directional characteristics. Based on Equation (2), by analyzing these drift magnitudes and their directional relationships, the magnitude and orientation of the magnetic field can be fully determined.

**Figure 9:**
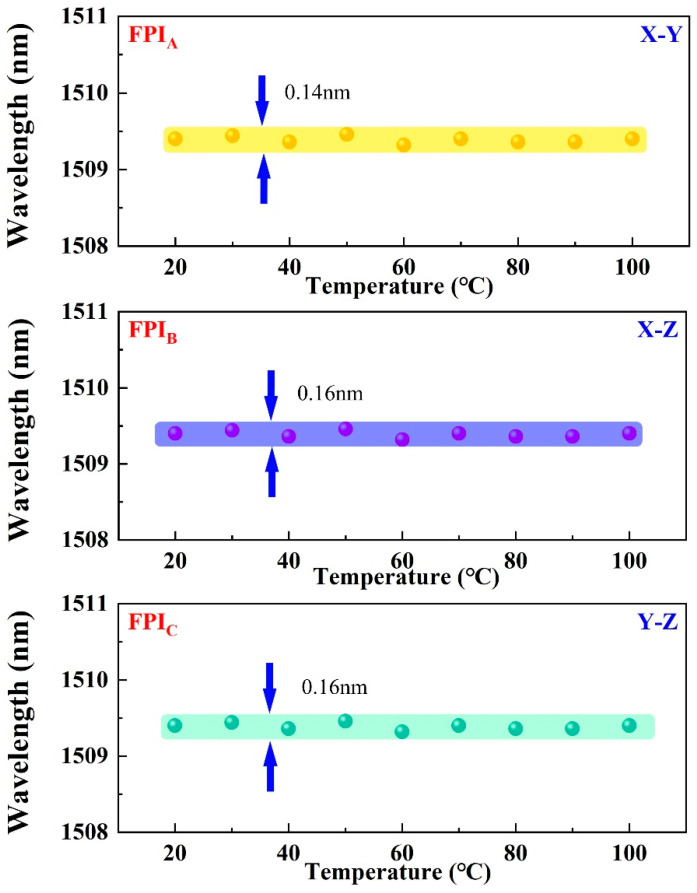
Temperature characteristic of the proposed sensor.

In addition, the temperature characteristic is also one of the most important parameters in magnetic field sensing. For the temperature characteristic test, the sensor is mounted on a temperature-controlled furnace, which is ramped up from room temperature (20 °C) to 100 °C with a step of 10 °C, as presented in Figure 9. For the Terfenol-D material, the magneto-striction with temperature is negligible in the linear section of the magneto-striction curve within a certain range of temperature [25]. Here, FPIs exhibit high temperature insensitivity after being bonded to the Terfenol-D block, and the total wavelength drifts of the FPIs are measured as below 0.14 nm, 0.16 nm and 0.16 nm over a temperature range of 20–100 °C. This additional advantage ensures that temperature-induced crosstalk exerts negligible influence on the performance of magnetic field sensing during measurements.

In this section, the ability for simultaneous determination of both the magnetic field direction and intensity of the prepared sensor is tested. Here, the prepared sensor is mounted on the 3D-printed fixture, which is positioned at a random angle at the center of the coil, while the current is adjusted to vary the magnetic field intensity. Under this circumstance, five groups of magnetic fields with distinct magnitudes and directions are configured, and the wavelength changes in the three FPIs are simultaneously monitored. Table 1 presents the wavelength drifts of the FPIs under various conditions as well as the magnitudes and directions of the true magnetic fields derived using Equations (3)–(5) and compares these derived values with the applied magnetic field. Here, the maximum relative error of magnetic field angle and intensity is obtained as 1.50% and 0.74%, respectively. Since each FPI only undergoes wavelength drift proportional to the magnetic field magnitude, the proposed sensor can only measure magnetic fields within the 0–90° range of both azimuth and polar angles. Future work will focus on optimizing the sensor’s configuration to enable full-range angular measurement capabilities.

**Figure 10:**
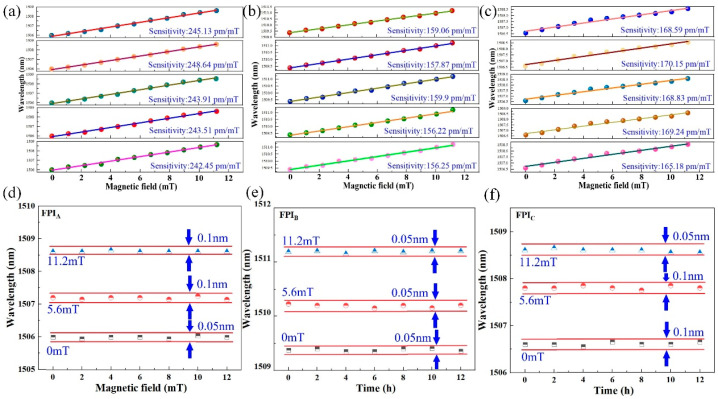
Repeatability tests of the (**a**) FPI_A_, (**b**) FPI_B_, and (**c**) FPI_C_. Stability tests of the (**d**) FPI_A_, (**e**) FPI_B_, and (**f**) FPI_C_.

Repeatability and stability are critical metrics for assessing sensor performance [26], and so this section evaluates the corresponding parameters of the proposed sensor. Firstly, the scalar magnetic field responses of each FPI are tested five times over five consecutive days, and the results are displayed in Figure 10a. Based on the experiments, through calculation the maximum deviations between the experimental and average sensitivity are obtained as 3.912 pm/mT for FPIA, 2.04 pm/mT for FPIB and 3.218 pm/mT for FPIC, corresponding to the repeatability errors of the three FPIs of 1.59%, 1.29% and 1.91. Meanwhile, the three FPIs are placed under three distinct magnetic field conditions, with the wavelength drift of each FPI measured per 2 h interval over a 12 h period. Under each magnetic field strength condition, the wavelength variations in the FPI in the X-Y, X-Z, and Y-Z planes are presented in Figure 10b. Herein, the maximum wavelength fluctuations of FPIs at 0, 5.6 mT and 11.2 mT are obtained as 0.1 nm, 0.1 nm and 0.1 nm, respectively. The wavelength oscillations of the FPIs are induced by the humidity-change noise effect. These results demonstrate that the sensor maintains excellent repeatability and stability over long-term operation, rendering it suitable for sustained high-performance applications.

To further clarify the performance of the proposed GMM-V-groove FPI sensor, a comprehensive comparison with other state-of-the-art fiber-optic and GMM-based magnetic sensors is presented in Table 2. As indicated, while most existing sensors, such as those based on MZI [27], MMI [28], or FBG [29], are limited to 1D vector measurements, our sensor achieves full 3D vector sensing capability. This allows for the simultaneous detection of magnetic field magnitude and its precise spatial orientation. Furthermore, compared to GMM-based FBG sensors [30], our proposed sensor exhibits a significantly higher sensitivity of 245.13 pm/mT. This remarkable enhancement is primarily attributed to the CO_2_ laser-modulated V-groove structure, which effectively reduces the longitudinal stiffness of the fiber and enhances the strain transfer efficiency from the GMM to the FPI. Additionally, compared to MF-based sensors that offer 2D sensing, our all-fiber FPI configuration provides superior structural robustness and a more compact design, making it more suitable for use.

## 5. Conclusions

In this article, a 3D vector magnetic field sensor is proposed and experimentally tested. Leveraging the high magneto-strictive properties of Terfenol-D, we integrated three FP structures, fabricated via identical methods, onto three mutually orthogonal planes of the Terfenol-D material. The devices exhibited distinct sensitivities in different directions, and experimental results show maximum sensitivities of 245.13 pm/mT, 159.06 pm/mT, and 168.59 pm/mT on the X-Y, X-Z, and Y-Z planes. Moreover, each FPI enables vector magnetic field measurement within its respective plane. For unknown magnetic fields, demodulating the real-time wavelength drift of the three FPIs allows quantification of fields with azimuth (0 < ϕ < 90°) and polar (0 < θ < 90°) angles. The proposed sensor achieves a compact structure, high sensitivity, and low cost, while maintaining excellent stability and repeatability during long-term operation. Additionally, it introduces a novel method for 3D magnetic field measurement and holds promise for magnetic field detection in precision instruments. 

## Figures and Tables

**Table 1:** Comparison of measured and applied magnetic field conditions.

	Applied Magnetic Field			Measured Results					
				Wavelength Shift			Measured Magnetic Field		
Test	ϕ	θ	Intensity	FPI_A_	FPI_B_	FPI_C_	ϕ	θ	Intensity
1	30	60	2.4 mT	430	160	200	29.7	59.6	2.39 mT
2	60	40	4.24 mT	340	370	540	59.1	40.2	4.23 mT
3	25	75	8.16 mT	1740	520	350	24.7	75.1	8.1 mT
4	85	30	9.64 mT	100	750	1400	84.9	29.8	9.69 mT
5	45	45	11.2 mT	1360	900	1320	45.4	45.3	11.21 mT

**Table 2:** Comparison of the sensing ability of the proposed sensor and previously reported magnetic field sensors.

Sensor Type	Measurement Range	Sensitivity	Dimensions	Ref
GMM + MZI	20.9–58 mT	1.33 MHz/mT	1D Vector	[27]
MF + MMI	47–60 mT	−0.865 dB/mT	1D Vector	[28]
GMM + FBG	0.121–0.261 T	1089.056 pm/T	1D Vector	[29]
MF + Ring-shaped Fiber	0–9 mT	2.402 dB/mT	2D Vector	[30]
GMM + V-groove FPI	0–11.2 mT	245.13 pm/mT	3D Vector	This work

## Data Availability

Data underlying the results presented in this paper are not publicly available at this time but may be obtained from the authors upon reasonable request.

## Abbreviations

The following abbreviations are used in this manuscript:

GMM	Giant magneto-strictive material
SMF	Single-mode fiber
HCF	Hollow-core fiber
MZI	Mach–Zehnder interferometer
